# Four types of vibration behaviors in a mole cricket

**DOI:** 10.1371/journal.pone.0204628

**Published:** 2018-10-10

**Authors:** Yaoko Hayashi, Jin Yoshimura, Derek A. Roff, Tetsuro Kumita, Akira Shimizu

**Affiliations:** 1 Department of Biological Sciences, Tokyo Metropolitan University, Hachioji, Tokyo, Japan; 2 Graduate School of Science and Technology, and Department of Mathematical and Systems Engineering, Shizuoka University, Hamamatsu, Japan; 3 Department of Environmental and Forest Biology, State University of New York College of Environmental Science and Forestry, Syracuse, New York, United States of America; 4 Marine Biosystems Research Center, Chiba University, Kamogawa, Chiba, Japan; 5 Department of Biology, University of California, Riverside, CA, United States of America; 6 Department of Physics, Tokyo Metropolitan University, Hachioji, Tokyo, Japan; Museum National d'Histoire Naturelle, FRANCE

## Abstract

Vibrational communication is known in some subterranean insects. Except for their use in sexual signaling, vibration behavior has rarely been reported. We report here four distinct types of substrate-based vibration behaviors in the mole cricket, *Gryllotalpa orientalis*, which are not associated with sexual signaling because of the occurrence of these behaviors in nymphs: (1) scraping with the forelegs; (2) foreleg taps (tapping with the forelegs); (3) palpal taps (tapping with the maxillary palpi); and (4) tremulation (back-and-forth movement of the whole body). Scraping is hypothesized to be used for the inspection of borrows. Foreleg taps are possibly informing nearby individuals of their presence, because it is never observed in solitary conditions. Palpal taps are rarely observed and its function is unknown. Tremulation is possibly related to avoidance of conspecific individual approaching and touching. The combination of the four vibration behaviors in the mole cricket may be unique among insects.

## Introduction

Vibration behaviors of various animals, from whales to insects, that act as communication signals have long attracted attention [1]. The method by which such vibrations are produced varies among animal species, depending on different sensory modalities and body structures. This type of communication provides information used in predator-prey interactions, food search, mate choice, intrasexual competition and maternal/brood social interactions [1–3]. Vibration behaviors should be common in subterranean animals, such as mole crickets and mole-rats. However, due to the difficulty of observing the underground behaviors, only a few studies reported on their vibration behaviors [4–7].

Among vibration behaviors, vibrational communication is most often reported in animals. To date, such communication has been recorded in more than 230,000 species of arthropods and vertebrates [8]. Claridge [9] stated “many groups of insects have developed specialized systems of sound production and associated receptors which are used in communication within and between species.” Most of them produce air-borne sounds for sexual communication [10, 11] (e.g., crickets, cicadas, katydids and bush crickets). Many of them, however, produce substrate-borne vibrations by rubbing or tapping particular parts of the body, such as legs, abdomens and maxillary palpi [10] (e.g., stoneflies [12] and anobiid beetles [13]). Virant-Doberlet and Čokl [7] reviewed studies of substrate-borne vibrations, exemplifying various production mechanisms, e.g., tremulation, drumming, tapping, tymbal and stridulation in as many as 96 insect families belonging to 16 orders. Substrate-borne signals are related to sexual behavior, alarm and defensive behavior [3, 7, 10, 14].

Mole crickets produce ‘calling songs’ or ‘courtship songs’ [15–28]. In some species, male mating calls are amplified by a “calling burrow” [15–23, 25, 27] (e.g., *Gryllotalpa australis*, *G*. *gryllotalpa*, *G*. *major*, *G*. *vineae*, *Scapteriscus borellii* (syn. *S*. *acletus*) and *S*. *vicinus*). In males of *S*. *vicinus*, tapping with forelegs on the soil is also associated with mating calls [28]. In the Japanese mole cricket *G*. *orientalis* (Burmeister), females also produce calling songs [29].

Other than communication behaviors, vibration behaviors and their functions are rarely investigated. For example, except for the calling songs produced by its wings, vibration behaviors are not so far known in *G*. *orientalis*. We here report the discovery of four distinct behaviors produced by substrate-based vibration in males, females and immatures of this species. We characterize these behaviors, by means of video, oscillogram and sonagram recordings and examine the effects of the presence of adjacent neighbors on these vibration behaviors. A combination of the four behaviors is reported for the first time not only in mole crickets but also in all insects. Interestingly, similar seismic vibrations, e.g., foot and head drumming and finger scratching, have been reported in fossorial mammals such as mole-rats, spalacid mole-rats and golden moles [4, 5]. It is thus expected that our studies will bring about intriguing results from the viewpoint of convergent evolution between insects and mammals with subterranean mode of life.

## Materials and methods

Worldwide, there are over 100 species of mole crickets (Gryllotalpidae, Orthoptera) [30]. One of their unique morphological characteristics is the fore tibia which has two to four large spine-like projections apically, two of which are tibial spurs. These are employed in digging. They are solitary and spend most of their life from egg to adult underground.

**Fig 1:**
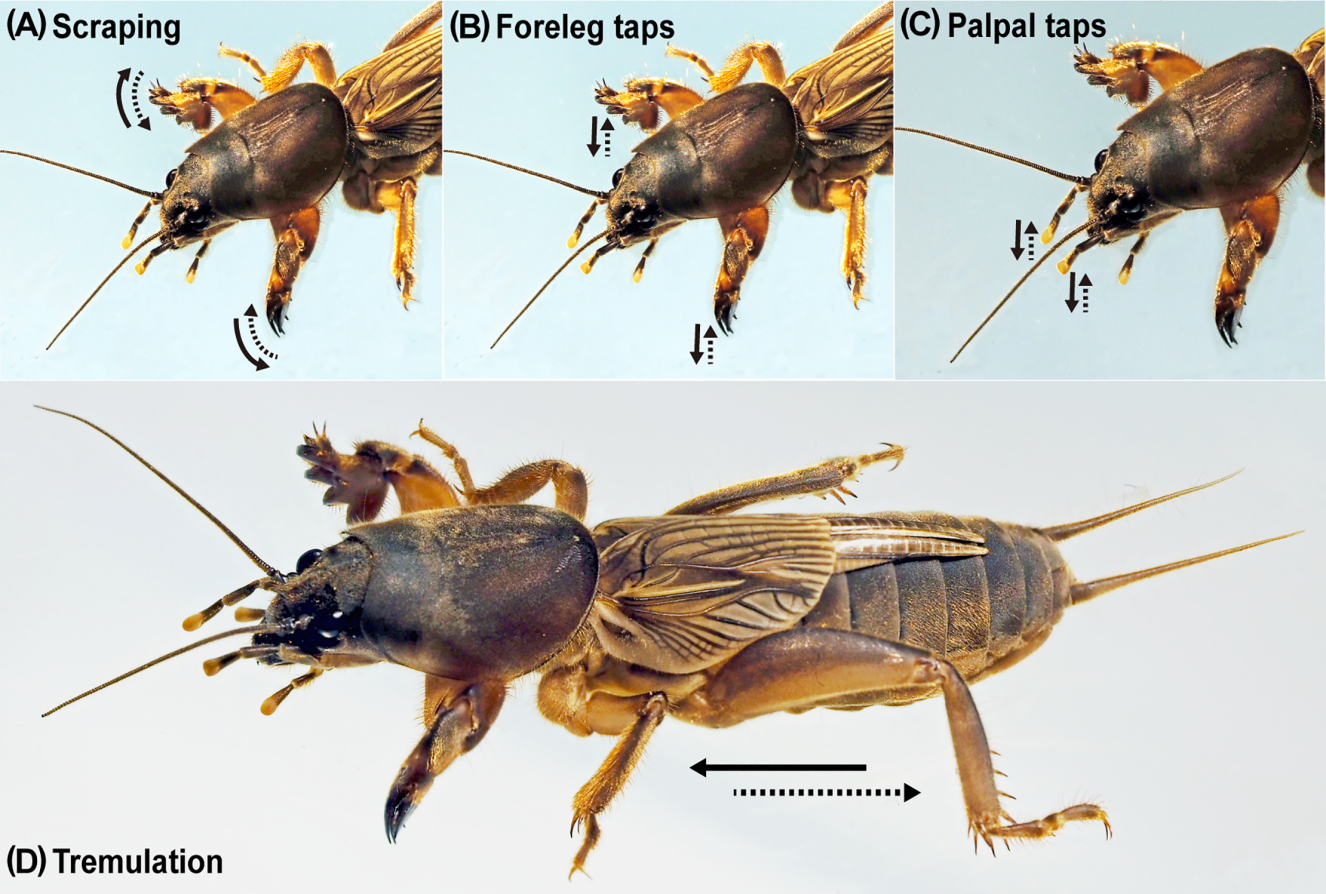
Movement of the body and appendages (indicated by arrows) used for four types of vibration behavior. (A) Scraping. (B) Foreleg taps. (C) Palpal taps. (D) Tremulation. (Photographed and illustrated by A. Shimizu.).

For the observations and experiments described here, we used the mole cricket *G*. *orientalis*, which is very common and the only species distributed throughout Japan. It is 3 cm in body length, with a cylindrical body covered with velvety pubescence (Fig 1D). The fore femur and tibia are broad and flattened laterally, suitable for digging burrows. Fundamentally the mole cricket is nocturnal. At night it actively feeds on food, songs and copulates in captivity, and flies toward lights in the field. From early summer to late summer, the female constructs egg chambers several times and lays 30–70 eggs within each of them [26]. After hatching, the nymphs remain in the chamber for about 10 days, and afterward disperse in separate directions, digging their own galleries. They molt eight times before becoming adults. The width of their burrows is almost equal to the span of the spread fore legs. Individuals were collected from Tokyo, Saitama (Honshu) and Kochi (Shikoku) Prefectures and bred through three generations. We tested 20 virgin males and females and 7–11 nymphs (some nymphs were not tested with all types of neighbors). Each individual was kept from its nymphal stage in a plastic container with moistened sphagnum moss at a temperature of 15–25°C, and fed with the larvae of midges, pieces of carrots, seeds of corns, oats and rice.

We designate the newly detected four types of vibration behavior as ‘scraping’, ‘foreleg taps’ (tapping with the forelegs), ‘palpal taps’ (tapping with the maxillary palpi) and ‘tremulation’. The term ‘scraping’ was originally used for larval scraping of their mandibles on the cell walls as they beg for food in vespid wasps [7, 31, 32]. In Plecoptera, scraping involves a short drag of the abdomen as it contacts the substrate, producing a raspy sound on resonant substrates [12]. We borrow the term for scraping the substrate with the forelegs in the Japanese mole cricket. Tapping of particular parts of the body on the substrate has been reported in various insect groups, such as Orthoptera, Psocoptera, Heteroptera, Coleoptera and Hymenoptera [7]. In Orthoptera, males of an American mole cricket (*Scapteriscus vicinus*) tap the soil with the forelegs [28] and an Australian cricket (*Balamara gydia*) produces percussion signals by tapping the reed it sits on, either with its abdomen (‘sternal taps’) or with the maxillary palps (‘palpal taps’) [33], although these behaviors are associated with courtship. Tremulation, which is the substrate vibration transmitted through the legs by moving the body back and forth without making actual body-substratum contact [9], has been recorded in many insect orders, such as Orthoptera, Plecoptera, Heteroptera, Neuroptera, Mecoptera, Diptera and Trichoptera [7].

**Fig 2:**
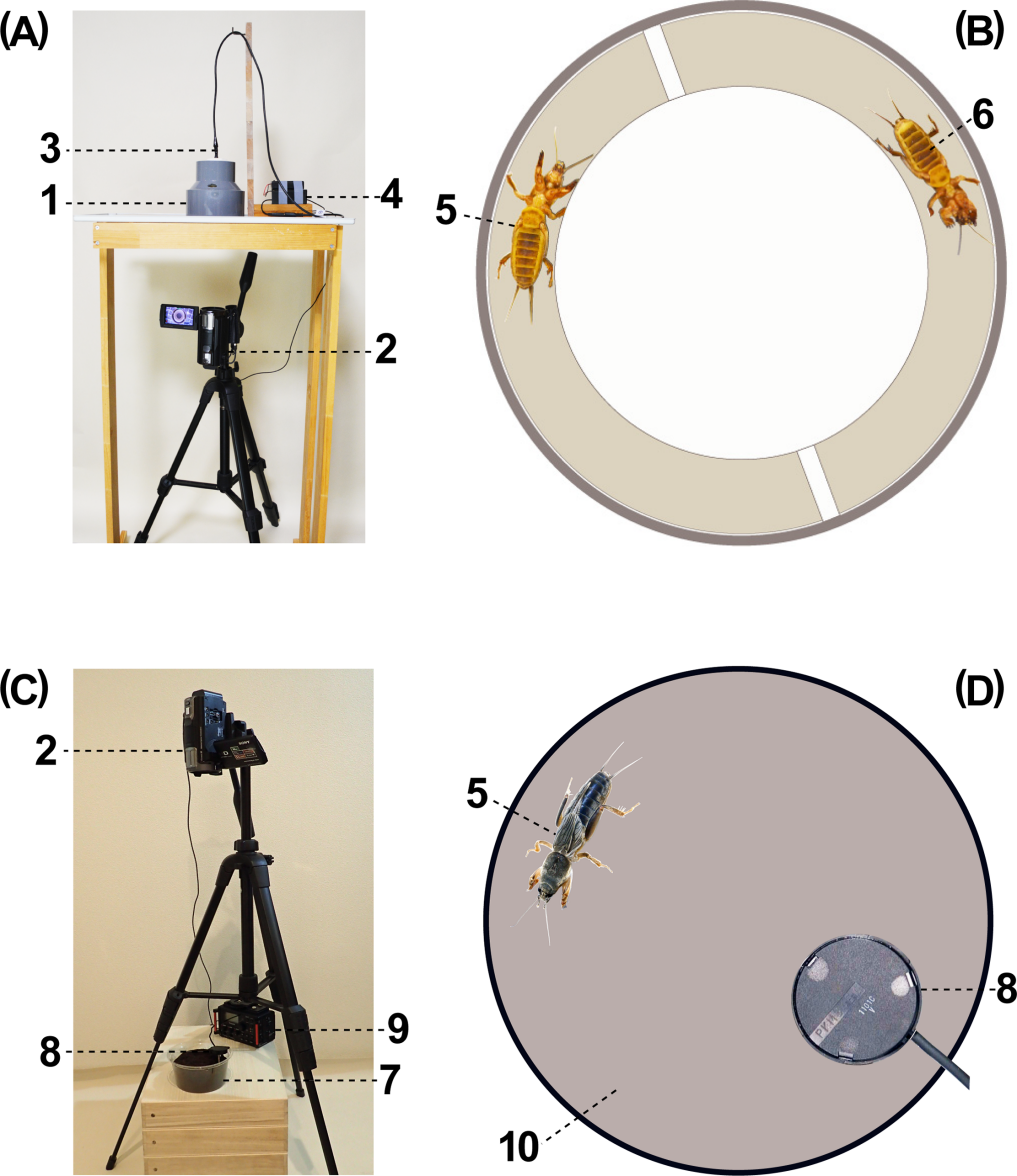
Experimental apparatus. (A) Photographic and sound-recording apparatus; (B) container used for recording scraping, foreleg taps, palpal taps and tremulation behaviors, seen from bottom; (C) photographic and sound-recording apparatus; (D) container used for recording scraping and digging behaviors, seen from above. 1, Container used for recording scraping, foreleg taps, palpal taps and tremulation behaviors; 2, video camera; 3, mini-microphone; 4, adaptor for microphone; 5, test individual; 6, neighbor; 7, container used for recording scraping and digging behaviors; 8, insect microphone; 9, amplifier for insect microphone; 10, soil floor. (Photographed and illustrated by Y. Hayashi.).

To record these behaviors, we prepared a cylindrical plastic container (12 cm in diameter, 11.5 cm in height) (Fig 2A). To facilitate visualization of the leg movement, a strip of white paper was put on the inner wall of the container. Another piece of paper was put inside the container along its periphery to provide a 1.5cm wide passage, which is almost equal to the span of the spread fore legs of an average-sized adult. Through the passage, mole crickets walked, touching both walls of the passage with their legs. This passage was divided in half by two pieces of white paper (Fig 2B). Test individuals (20 males, 20 females and 20 nymphs) were randomly selected out of 125 individuals we were rearing. We put a test individual in one partition of the passage and, when necessary, another individual (neighbor) in the other partition. The container was placed on a transparent board of PET (polyethylene—terephthalate), through which we recorded the behavior with a video camera (SONY HDR-CX720) located underneath. We also installed a small microphone on the container ceiling to record the vibrational sound of the mole cricket simultaneously with the video recording at a temperature of 19–21°C. The records of the sound were converted to oscillograms and sonagrams by using the software Onseikobo SP4WIN Yuragi (NTT Advance Technology).

First, for the observation and recording of a solitary individual, we put a mole cricket (male, female or nymph) in one partition of the passage and recorded its behavior for 45 minutes. From this recording we counted the occurrence number of each behavior (scraping, foreleg taps, palpal taps and tremulation) by analyzing 40 minutes of the audio-visual recording, discarding the initial five minutes.

Next, to test the effects of a neighbor, 10 minutes after placing the first cricket, we put another mole cricket (male, female or nymph) in the other partition of the passage, recorded the behavior of the subject individual for a further 45 minutes and, as before, analyzed the 40 minutes of the recording, excluding the first five minutes. Over a four day period, each individual was tested alone, with a male, with a female, and with a nymph.

To compare scraping behavior with digging one, we use a container with soil (Fig 2C and 2D). For this, we prepared a plastic container (10 cm in diameter, 5 cm in depth), put fine-grained clayey soil on the bottom 2 cm in depth lest the mole cricket should scrub the bottom with the fore legs. After drying the soil, we further added chernozem (black earth) soil 0.5 cm in depth and daubed black earth soil on the inner wall of the container brimfully. Mole crickets under these conditions exhibited both ‘scraping’ and ‘digging’ behaviors. We installed a microphone (INS-M, NARIKA) on the tip of a nail (5 cm long) stood in the soil of the container to record the substrate vibration of the mole cricket simultaneously with the video recording. The records of the vibration were converted to oscillograms and sonagrams as stated above.

In our preliminary experiments, we observed tremulation behavior when an individual was in the presence of an individual of the same species. In this experiment, we used a transparent plastic container (6 or 10 cm in diameter, 1 or 10 cm in height) with or without sphagnum moss. Into this container, we first put an adult mole cricket and then an adult individual of the same species (n = 257). Under similar condition, we also confirmed whether nymphs performed the four types of behavior, by putting a nymph first and then a male or female adult or a nymph in a container (n = 276). We complemented the results of the experiments with those of the preliminary observations.

## Results

**Fig 3:**
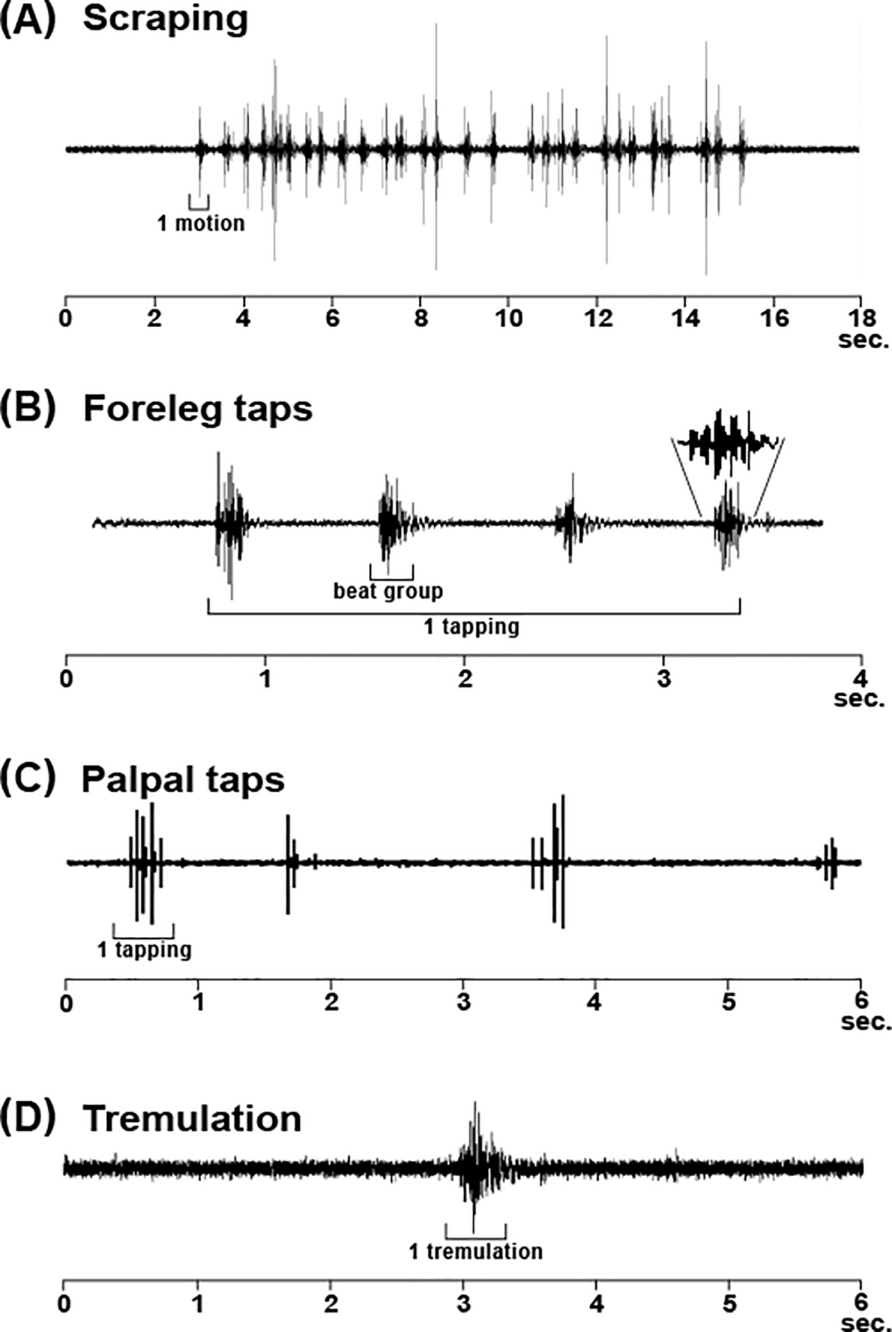
Osillograms of four oscillation behaviors. Scraping; (B) foreleg taps; (C) palpal taps; (D) tremulation. In scraping, a motion constitutes many beats. A single tapping of foreleg taps constitutes several beat groups, one of which contains 5–7 beats (pulses). A single tapping of palpal taps constitutes 2–5 beats. A single tremulation constitutes several motions. Status of test individual, date and start time at each experiment is as follows: [A: adult female, 6 March 2013, 12:27]; [B: adult male, 20 February 2013, 16:02]; [C: adult female, 6 March 2013, 12:28]; [D: adult male, 27 February 2013, 14:00]. Temperature at the recordings is 19–21˚C.

Our analyses of the movies, oscillograms and sonagrams revealed that four distinctive types of vibration behavior occur in adults: (1) scraping, (2) foreleg taps, (3) palpal taps and (4) tremulation (Figs 1 and 3; Tables 1–3; S1–S5 Movies). Nymphs also exhibited all of the behaviors in the preliminary observations (scraping: 106/276 individuals; foreleg taps: 7/276 individuals; palpal taps: 4/276 individuals; tremulation: 11/276 individuals), although foreleg taps and palpal taps were not found in the present experiment (Table 1).

**Table 1:** The occurrence number (average number ± standard deviation) of four behaviors, scraping, foreleg taps, palpal taps and tremulation in 40 minutes recordings.

Subject	Solitary	Neighbors (non-solitary)		
		Male	Female	Nymph
**Scraping**				
Male	583.5±751.5 (n = 20)	370.4±336.6 (n = 20)	340.5±470.7 (n = 20)	376.6±593.5 (n = 20)
Female	210.5±218.8 (n = 20)	32.2±88.9 (n = 20)	89.7±145.3 (n = 20)	134.2±246.8 (n = 20)
Nymph	145.9±176.0 (n = 11)	29.4±31.7 (n = 7)	26.0±37.8 (n = 11)	119.4±175.9 (n = 7)
**Foreleg taps**				
Male	0±0 (n = 20)	1.2±4.3 (n = 20)	0.2±0.7 (n = 20)	0±0 (n = 20)
Female	0±0 (n = 20)	0.3±1.1 (n = 20)	0±0 (n = 20)	0±0 (n = 20)
Nymph	0±0 (n = 11)	0±0 (n = 7)	0±0 (n = 11)	0±0 (n = 7)
**Palpal taps**				
Male	0.3±0.6 (n = 20)	0.1±0.3 (n = 20)	0.1±0.4 (n = 20)	0±0 (n = 20)
Female	0.3±0.9 (n = 20)	0.4±1.3 (n = 20)	0±0 (n = 20)	1.7±4.9 (n = 20)
Nymph	0±0 (n = 11)	0±0 (n = 7)	0±0 (n = 11)	0±0 (n = 7)
**Tremulation**				
Male	0.2±0.4 (n = 20)	1.7±2.9 (n = 20)	1.3±1.5 (n = 20)	0.7±1.4 (n = 20)
Female	0.5±1.1 (n = 20)	1.1±2.0 (n = 20)	0.6±1.1 (n = 20)	0.4±1.0 (n = 20)
Nymph	0.3±0.6 (n = 11)	0.1±0.4 (n = 7)	0±0 (n = 11)	0±0 (n = 7)

**Table 2:** Two-way ANOVA of scraping behavior as a function of the type of individual (Type* = male, female, nymph) and individual status (Status = solitary, with a male, female or nymph neighbor).

	Sum. Sq	Df	F value	Pr (>F)
Type	47.68	2	7.746	0.0005969
Status	94.76	3	10.263	2.92E-06
Type* status	69.34	6	3.755	0.0015303
Residuals	541.66	176		

Two-way ANOVA of scraping behavior as a function of function of the type of individual (Type = male, female, nymph) and individual status (Status = solitary, with a male, female or nymph neighbor).

**Table 3:** Two-way ANOVA of scraping behavior as a function of the difference log(Y_solitary_+1)-log(Y_Neighbor_+1) (Type* = male, female, nymph).

	Sum Sq	Df	F value	Pr (>F)
Type	5.154	2	3.0743	0.049548
Neighbour	7.058	2	4.2104	0.016881
Type* neighbor	8.136	4	2.4267	0.05106
Residuals	110.643	132		

### Scraping

The mole cricket spread the forelegs anterolaterally, quickly moved them outwardly and in an arc, rubbing the substrate with the apical projections of the tibiae, and then returned them to their original positions (Fig 1A; S1 and S5 Movies). This reciprocation formed a single motion, lasting 0.1–0.3 seconds (Figs 1A and 3A; S1 and S2 Figs), during which time the cricket stayed at the same location.

**Fig 4:**
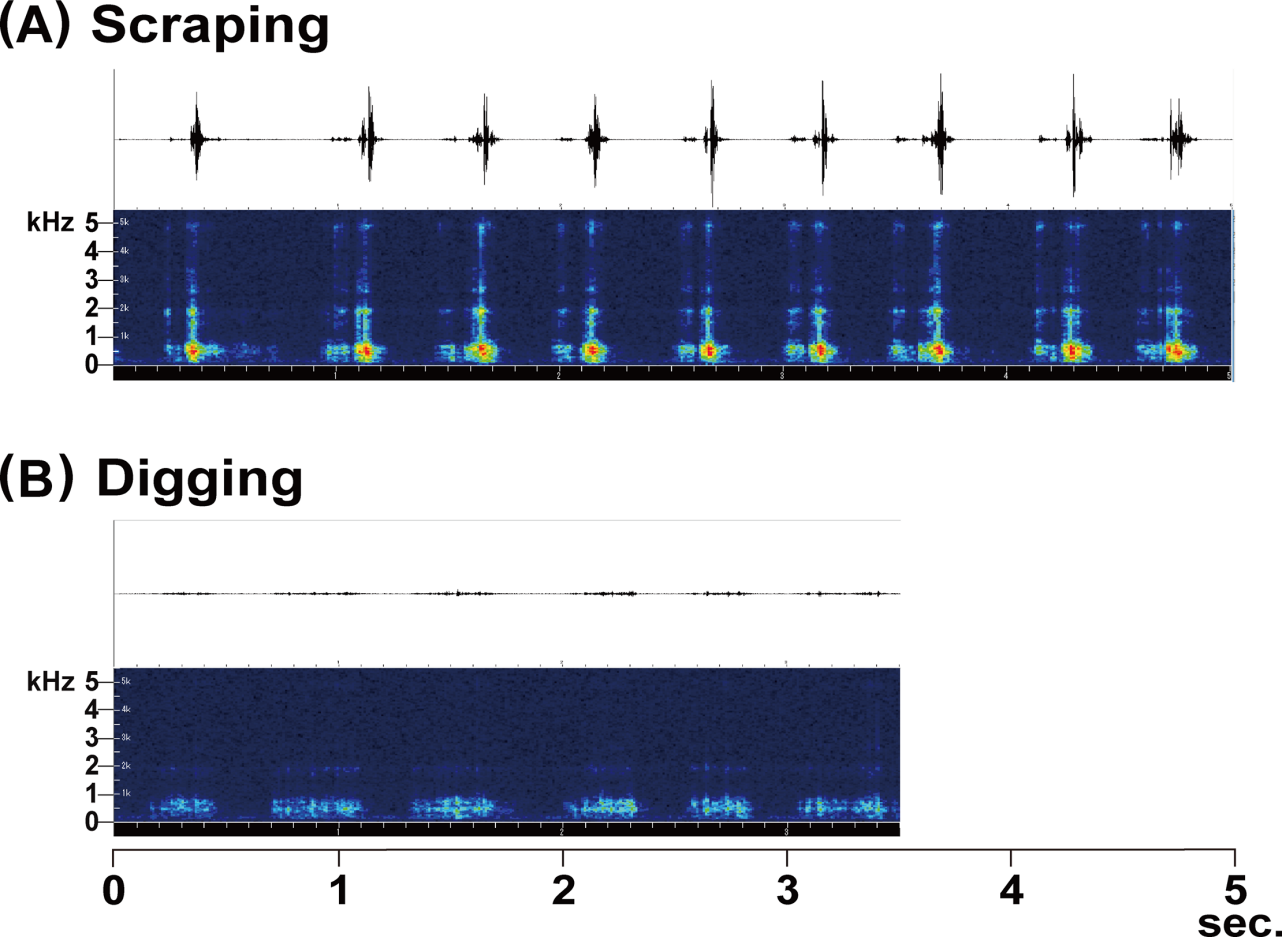
**Oscillograms (upper) and sonagrams (lower) of two oscillation behaviors.** (A) Scraping; (B) digging. Status of test individual, date and start time at each experiment is as follows: [A: adult female, 11 February 2017, n/a.]; [B: adult female, 11 February 2017, n/a.]. Temperature at the recordings is 20.5°C.

From the vibratory waveform of scraping it is apparent that the interval of the wave peaks was 0.1–0.8 seconds (Fig 3A). The vibration impulse of 0–1 kHz was very strong, representing its maximum value at about 0.5 kHz, together with weak vibration pulse in 1–5 kHz (Fig 4A; S1 and S2 Figs). This suggests that a mechanical force was momentarily applied to the substrate. One scraping behavior, comprising one to many motions, was continued for 1–67 seconds.

Scraping was similar to digging except that, in the latter behavior, the mole cricket slowly moved the forelegs inward and outward and pushed the soil outward, thereby creating a passage in front of the individual (S5 and S6 Movies). The vibration impulse of digging appears weak in 0–1 kHz and the higher frequency components are lacking (Fig 4B; S1 and S2 Figs). The waveform of digging (Fig 4B) suggests that mechanical force was consecutively applied to the substrate for less than 1 second. One digging behavior was continued for 5–20 seconds.

We statistically analyzed the occurrence number of scraping as a function of the type of individual (Type = male, female, nymph) and individual status (Status = solitary, with a male, female or nymph neighbor) with a two-way ANOVA (Tables 1–3). For this analysis, we only used nymphs (n = 7) that were tested against all three types of neighbor. To better satisfy normality we transformed the observations (y) using log(y+1). Both Type and Status and the interaction between them were significant (Table 2). Because the same individual was tested against all combinations of neighbors, we also tested for variation using the difference log(Y_solitary_+1)-log(Y_Neighbor_+1). This analysis gave a marginally non-significant effect of the interaction between Type and Neighbor but significant additive effects of these (Table 3). Thus the amount of scraping is dependent on both the type of individual and its neighbor, possibly suggesting some form of communication.

### 2) Foreleg taps

The mole cricket spread the forelegs anterolaterally and tapped the substrate with the fore tibial projections (Fig 1B; S2 Movie). We defined a single beat group as a series of motions. One tapping behavior comprises a consecutive series of 3–9 beat groups (groups of pulses); the intervals between two consecutive beat groups were rather constant, lasting 0.7–1 second. We analyzed the occurrence number of tapping and its vibratory waveform (Fig 3B). Tapping was never observed in the absence of a neighbor in all preliminary and present experiments, although it occurs infrequently in the presence of a neighbor (Table 1).

### 3) Palpal taps

The mole cricket spread the maxillary palpi anterolaterally and tapped the substrate several times with the apical swollen part, one of the palpi moving up and down or both palpi moving alternately (Fig 1C; S3 Movie). As in tapping, we defined a consecutive series of motions as one tapping behavior and investigated the occurrence number of tapping and the vibratory waveform. One tapping comprised 2–10 beats. The sound of the tapping was faint and irregular in rhythm (Fig 3C). Palpal taps were very infrequent in all conditions (Table 1).

### 4) Tremulation

The mole cricket supported the body with the mid and hind legs, touching them to the underside and sidewalls of the passage, and spread the forelegs anterolaterally off the substrate. The cricket then moved the body back and forth quickly several times (Fig 1D). We categorized this behavior from start to end as one tremulation behavior. It took about 0.5 to 1.8 seconds. (Fig 3D), during which the cricket moved the body back and forth five to ten times (Table 1). The average occurrence number of tremulation of both male and female was low in the absence or presence of a neighbor. We did not observe this behavior in solitary individuals but only when a mole cricket was approached or touched by an individual of the same species (26/257 individuals; S4 Movie).

## Discussion

We discovered four distinct behaviors based on contact with the substrate in *G*. *orientalis*: scraping, foreleg taps, palpal taps and tremulation. Both male and female adults performed these behaviors (Table 1). The occurrence of these four types of behaviors in nymphs suggests that these are not specifically associated with mate recognition or mating. Note that the mole cricket uses its unique fore tibial projections for scraping and foreleg taps.

The average occurrence number of scraping (per 40 minutes) was different between subjects: when they are solitary, it is higher in males, then in females and lowest in nymphs (Table 1). The amount of scraping decreased significantly in the presence of a neighbor (Tables 1 and 2). It is also a function of the type of neighbor, specifically in females and nymphs (Table 2). Against male neighbors, females reduced the average number of scraping from 210.5 to 32.2 (Table 1). Against adult neighbors, nymphs reduced from 145.9 to 29.4 (male neighbors) and 26.0 (female neighbors) (Table 1). These reductions of scraping suggest that the mole cricket was avoiding being noticed by its neighbor. These suggest that the primary function of scraping would be inspection of burrows (both ground and walls in front of a subject). However, females and nymphs reduce vibrations for burrow inspection probably to avoid copulation attempts by males and cannibalism by adults, respectively.

Scraping and digging are similar in terms of foreleg movement but differ in the mechanical force applied against the substrate. In scraping, the mole cricket applies momentary pressure on the substrate, hence strong vibratory impulse in low-frequency (0–1 KHz) (Fig 4A; S1 and S2 Figs), but does not move the soil. In digging, the cricket adds consecutive pressure to the substrate for less than one second, emitting only weak vibratory impulse in low-frequency (0–1 KHz) (Fig 4B; S1 and S2 Figs), and moves the soil sideways to make a passage. The movement of the forelegs is also different between them. Scraping is only left/right movements. In contrast, digging is from left/right to up/down movements, because the frontal body is twisted up to 90 degrees angles. Note that, seen from the head of the insect, these movements are always left/right. We also note that scraping is associated with no body movement, while digging with progressive movements of the whole body. Thus scraping is evidently different from digging in their expressions in sonagrams and the body and fore movements.

Foreleg taps and palpal taps did not occur frequently (per 40 minutes). Foreleg taps was found only when there was another individual nearby (Table 1) and thus may function to check for the presence of an individual in a nearby burrow. Because tremulation occurred more frequently when an individual of the same species approached, its signals may be associated with defensive behavior. Tremulation often occurs before an actual contact with these neighbors; the mole cricket may sense their approach by the vibration of their movements, such as footsteps. Thus tremulation may be related to defensive behavior.

Vibration behaviors, such as tremulation and tapping with the abdomen, hind tarsi (heels) and maxillary palpi are known in many above-ground insects [2, 7, 10]. For example, palpal taps on the reed it sits on are reported for the Australian *Balamara gydia* (Orthoptera: Trigonidiidae) [33], although their function is unknown. These vibration behaviors, however, have seldom been reported in underground insects [2, 7, 10]. Among underground/semi-underground mammals, drumming behavior using the feet, head and teeth has been studied in at least 32 species of 11 families, including fossorial and semi-fossorial rodents [4]. The function of this behavior is unknown in almost all the cases, but it may function as an alarm signal to conspecifics and/or to predators and as cues to both detect and localize prey [6]. Some of the functions of the vibration behaviors in the mole cricket may be similar to those in underground mammals. Probably the combination of the four behaviors in the Japanese mole cricket has evolved in relation to its subterranean mode of life. Future studies are needed to elucidate functions of substrate-based vibration behavior common to fossorial and semi-fossorial animals from insects to mammals.

## Supporting information

S1 Fig (TIF)

S2 Fig (TIF)

S1 Movie Scraping. (AVI)

S2 Movie Foreleg taps. (AVI)

S3 Movie Palpal taps. (AVI)

S4 Movie Tremulation. (AVI)

S5 Movie Scraping on soil. (AVI)

S6 Movie Digging on soil. (AVI)
