# Supplementary figures and images for: Four types of vibration behaviors in a mole cricket
Source: PLoS One. 2018 Oct 10;13(10):e0204628. doi: 10.1371/journal.pone.0204628 (PMC6179226; doi:10.1371/journal.pone.0204628)

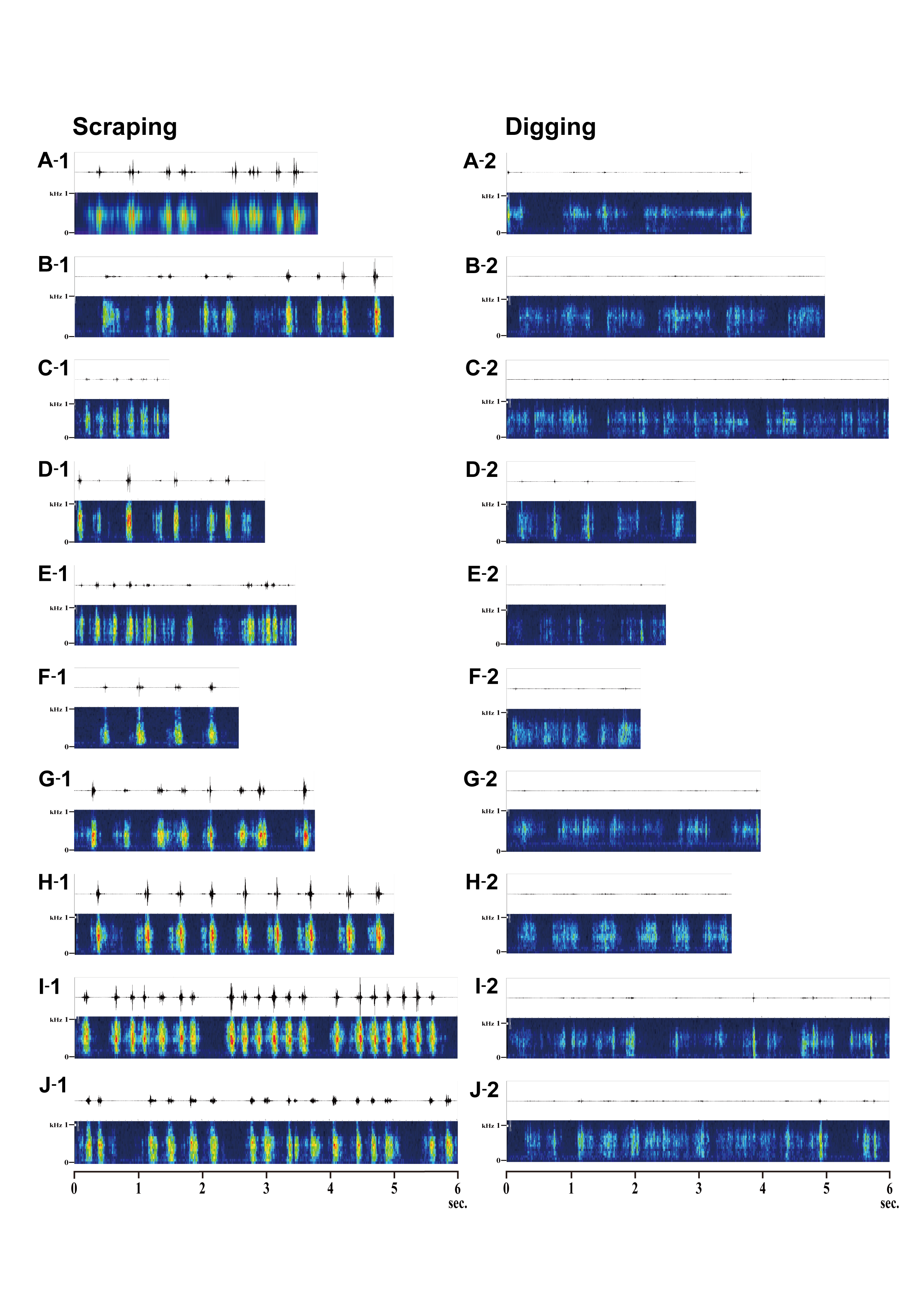

Supplement: S1 Fig — (TIF) [file pone.0204628.s001.tif]

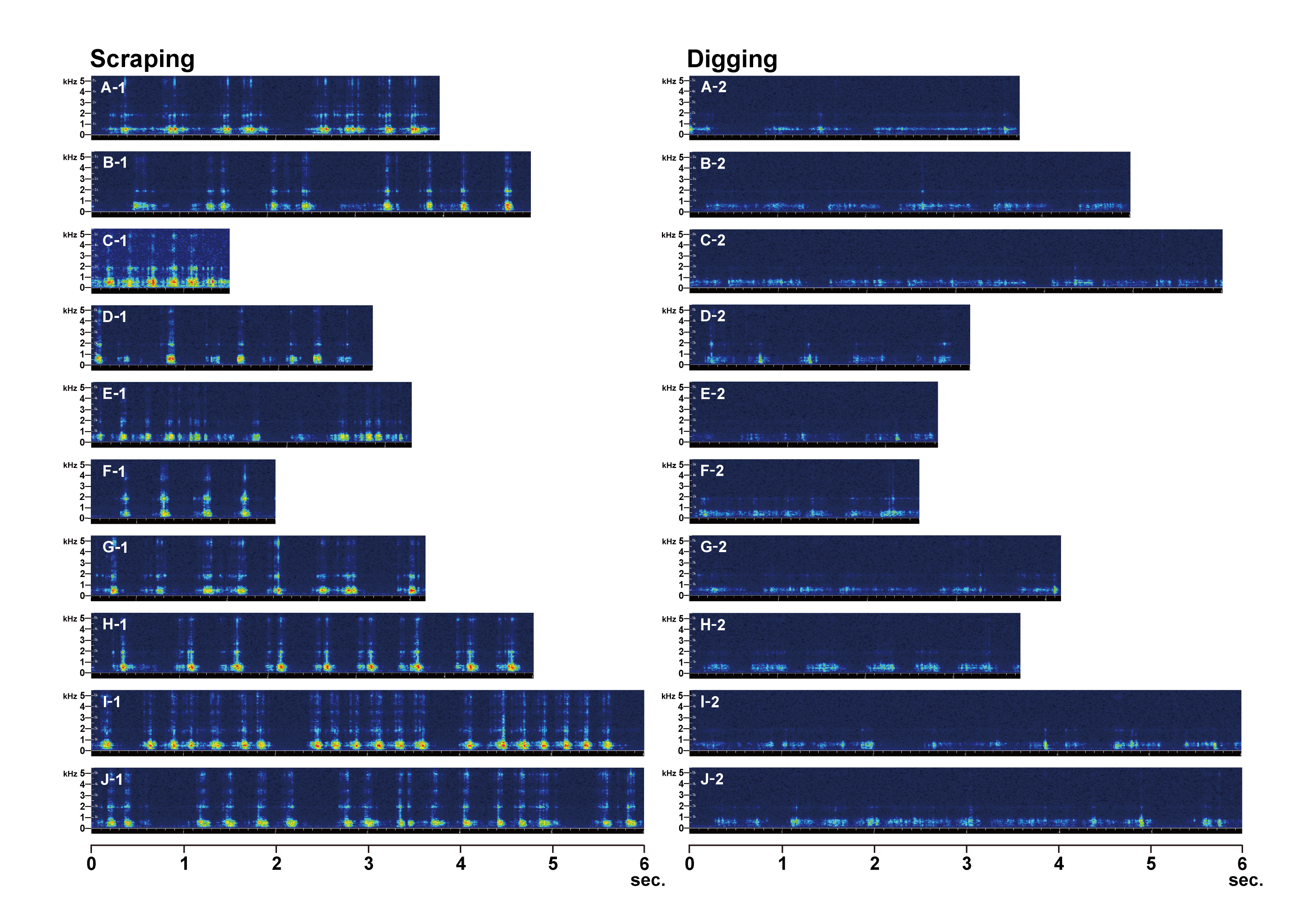

Supplement: S2 Fig — (TIF) [file pone.0204628.s002.tif]
